# Generating Coherent Raman Scattering Using a Molecular Optomechanical Cavity

**DOI:** 10.1021/acs.nanolett.5c04075

**Published:** 2025-11-07

**Authors:** Jian Huang, Dangyuan Lei, Zhedong Zhang

**Affiliations:** † Department of Physics, 121844 City University of Hong Kong, Kowloon, Hong Kong SAR; ‡ Department of Materials Science and Engineering, 53025 City University of Hong Kong, Kowloon, Hong Kong SAR; ¶ City University of Hong Kong, Shenzhen Research Institute, Shenzhen, Guangdong 518057, China

**Keywords:** Coherent Raman scattering, Coherent anti-Stokes Raman scattering, Stimulated Raman scattering, Molecular optomechanics, Collective enhancement effect

## Abstract

Coherent Raman scattering, e.g., coherent anti-Stokes Raman scattering (CARS) and stimulated Raman scattering (SRS), has emerged as a powerful tool for label-free molecular imaging in biological and biomedical systems. Here we develop an optomechanical approach for coherent Raman spectroscopy with a focus on the CARS and SRS. The results show that the Raman cross section can be significantly enhanced by increasing the pump strength. It turns out that the CARS signal is robust to the external temperature, yielding an order of magnitude amplification due to √*N* collectivity. We further find that the power spectrum of the emission is dominated by the SRS process. The SRS signal presents an anti-Stokes component appreciably stronger than the Stokes one. Our work suggests a new scheme for generating coherent Raman signals with enhanced stability and signal-to-noise ratio, which would be beneficial for molecular spectroscopy.

## Graphical abstract

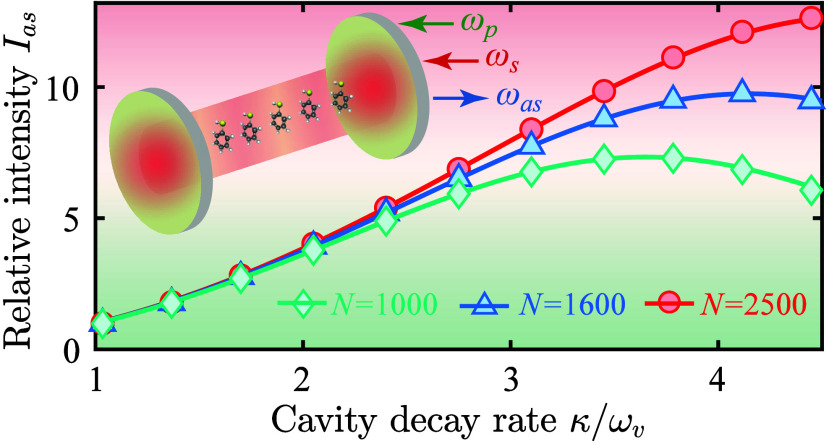

Raman spectroscopy,^1,2^ as an incredible fingerprint of the molecular structure and compounds, has been widely used in various fields of research including physics, chemistry, materials science, medicine, etc.^3–6^ However, the off-resonant nature makes spontaneous Raman scattering intrinsically weak. To overcome this, the coherent Raman techniques were developed,^7^ thanks to the glory of the nonlinear optical spectroscopy using laser pulses.^8^ Such an advancement led to the schemes of CARS^9–12^ and SRS,^13,14^ which have shown the power of probing reaction kinetics and species characterization of molecules. Nevertheless, the SRS signal is normally extremely weak on top of the probe field one is measuring. This led to a bottleneck in its application. The CARS scheme, due to the label-free, noninvasive, and highly sensitive features, has been broadly developed in various fields including imaging,^15–23^ chemical sensing,^24–28^ and spectroscopic techniques.^29–34^

Molecular optomechanics,^35–48^ originating from the quantum mechanical description of Raman scattering of molecules in plasmon cavities, provides a reliable theoretical tool to explore spectroscopy. Unlike traditional optomechanics,^49^ molecular optomechanics extends the scope from low-frequency MHz mechanical oscillators to the high-frequency THz molecular vibrations. Furthermore, it brings optomechanics closer to achieving single-photon strong coupling by confining molecules within ultrasmall volume plasmonic cavities. Due to these advantages and the accurate description of coherent photon–vibration interaction given by optomechanics, molecular optomechanical systems present a highly promising platform for investigating quantum effects related to Raman scattering as well as various intriguing phenomena, such as strong nonlinearities of the emitted signal,^37^ the Raman-induced optical spring effect,^35,45^ higher-order Stokes scattering,^36^ complex Raman photon correlations,^39^ and THz frequency up-conversion.^50–53^ In this context, the generation of coherent Raman spectroscopy in molecular optomechanical systems has emerged as a captivating and pivotal research topic. However, the appropriate theoretical approach for this purpose remains an open question.

In this Letter, we develop a molecular optomechanical scheme for coherent Raman spectroscopies, i.e., CARS and SRS. A microscopic theory is developed, making use of the optomechanical coupling coupling to the molecular vibrations. The results show that a significant optical spring effect caused by Raman scattering can be achieved by employing a strong pump. The results also show that the CARS signal is robust to environmental noise and exhibits an order of magnitude amplification for the input Stokes signal, attributed to collective enhancement. We further investigate the SRS signal, which exhibits a nonlinear dependence on the number *N*. Additionally, we demonstrate an anomalous enhancement of the anti-Stokes component with temperature, even surpassing that of the Stokes component. Our work proposes a robust framework for exploring coherent Raman spectroscopy in optomechanical systems.

We first consider a cavity–molecule system consisting of an optomechanical cavity and *N* molecules [see Figure 1(a)]. The optomechanical cavity can be implemented using a nanoparticle-on-mirror configuration,^51–54^ where the vibrational modes of *N* molecules (with frequency ω_*v*_) are coupled to the cavity mode (ω_*c*_). A pump field (frequency ω_*p*_ and amplitude *ε*_*p*_) and a Stokes field (ω_*s*_ and *ε*_*s*_) are input into the optomechanical cavity. In a rotating frame at frequency ω_*p*_, the Hamiltonian of this system reads (ℏ = 1)

1 $$H=\Delta_{c}a^{†}a+\overset{N}{\underset{j=1}{\sum }}\left[\omega_{v}b_{j}^{†}b_{j}+ga^{†}a\left(b_{j}^{†}+b_{j}\right)\right]+i\left(\varepsilon_{p}a^{†}+\varepsilon_{s}e^{-i\delta_{s}t}a^{†}-H.c.\right)$$

where *a* (*a* ^†^) and *b*_*j*_ (*b*_*j*_^†^) are the annihilation (creation) operators of the cavity mode and the vibrational mode of the *j*th molecule, respectively. Δ_*c*_ = ω_*c*_ – ω_*p*_ (δ_*s*_ = ω_*s*_ – ω_*p*_) is the detuning between cavity (Stokes) field and pump field, while *g* is the vacuum optomechanical coupling strength. Furthermore, $\varepsilon_{p}=\sqrt{2\kappa P_{p}/\hbar \omega_{p}}$ and $\varepsilon_{s}=\sqrt{2\kappa P_{s}/\hbar \omega_{s}}$ , where $P_{p}$ ( $P_{s}$ ) is the power of the pump (Stokes) field and κ is the cavity decay rate.

**Figure 1:**
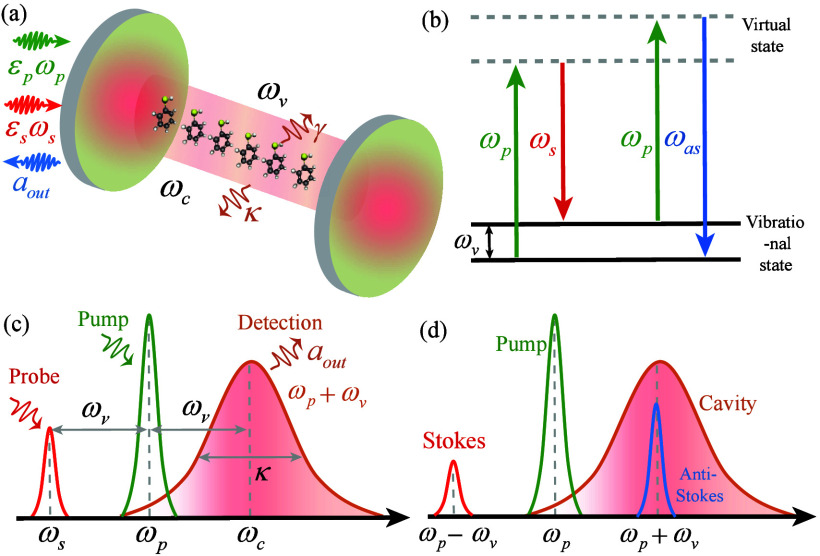
(a) The cavity–molecule system consisting of *N* molecules (with vibrational frequency ω_*v*_ and decay rate γ) optomechanically coupled to the cavity mode (ω_*c*_ and κ). A pump field (with frequency ω_*p*_ and amplitude *ε*_*p*_) and a Stokes field (ω_*s*_ and *ε*_*s*_) are introduced to drive the cavity. (b) Energy level diagram of the CARS process. The molecular vibrational coherence is prepared by the pump (ω_*p*_) and Stokes (ω_*s*_) fields. The CARS signal (ω_*as*_) is generated by the pump field (ω_*p*_) scattering off of the molecular vibration (ω_*v*_). (c) Scheme of CARS signal. Here the cavity field and the Stokes field are resonant with the first anti-Stokes and Stokes sideband of the pump field (i.e., ω_*c*_ = ω_*p*_ + ω_*v*_ and ω_*s*_ = ω_*p*_ – ω_*v*_), respectively. The output CARS signal (*a*_out,–_) with frequency 2ω_*p*_ – ω_*s*_ (ω_*p*_ + ω_*v*_) is generated via the optomechanical interaction. (d) Schematic of the Raman spectrum. The anti-Stokes process (blue) is selectively enhanced over the Stokes process (red) by the cavity resonance (brown line) under the red-detuned resonance case (Δ = ω_*v*_).

Due to the natural degeneracy of the vibrational modes, we introduce the collective operator $B=\overset{N}{\underset{j=1}{\sum }}b_{j}/\sqrt{N}$ with [*B*, *B* ^†^] = 1, where the Hamiltonian ([Disp-formula eq1]) becomes

2 $$H=\Delta_{c}a^{†}a+\omega_{v}B^{†}B+\sqrt{N}ga^{†}a\left(B^{†}+B\right)+i\left(\varepsilon_{p}a^{†}+\varepsilon_{s}e^{-i\delta_{s}t}a^{†}-H.c.\right)$$

The CARS signal can be analyzed via the quantum fluctuations; then we write the operator *o* ∈ {*a*, *B*} as a sum of the steady-state mean value and the quantum fluctuation, i.e., *o* = ⟨*o*⟩_*ss*_ + *δo*.^55^ The linearized quantum Langevin equations are

3 $$\begin{array}{c} \delta ȧ=-\left(i\Delta +\kappa \right)\delta a-i\sqrt{N}G\left(\delta B^{†}+\delta B\right)+\varepsilon_{s}e^{-i\delta_{s}t}+\sqrt{2\kappa }a_{\mathrm{in}},\\ \delta Ḃ=-\left(i\omega_{v}+\gamma \right)\delta B-i\sqrt{N}\left(G^{*}\delta a+G\delta a^{†}\right)+\sqrt{2\gamma }B_{\mathrm{in}} \end{array}$$

where Δ and γ are the normalized detuning and the vibrational decay rate, respectively; *G* = *g*⟨*a*⟩_ss_ is the linearized optomechanical coupling strength; *a*_*in*_ and *B*_*in*_ are the noise operators. *ε*_*s*_ is a weak field, of much lower intensity than the pump field *ε*_*p*_. The mean values ⟨*a*⟩_*ss*_ = *ε*_*p*_/(*iΔ* + κ) and

4 $$\langle B\rangle_{\mathrm{ss}}=\frac{-ig\sqrt{N}\varepsilon_{p}^{2}}{\left(i\omega_{v}+\gamma \right)\left(\kappa^{2}+\Delta^{2}\right)}$$

which gives a measure of collective molecular coherence and is a key to generate CARS.

To solve eqs 3, we use the ansatz^58–61^ ⟨*δo*⟩ = *o*_+_ *e* ^–*iδ*_*s*_ *t*^ + *o*_–_ *e* ^*iδ*_*s*_ *t*^, where *o*_+_ and *o*_–_ represent the values of positive- and negative-frequency components, respectively.^55^ As we considered a rotating frame at ω_*p*_, the cavity field in the initial frame is

5 $$\left\langle a\right\rangle =\langle a\rangle_{\mathrm{ss}}e^{-i\omega_{p}t}+a_{+}e^{-i\omega_{s}t}+a_{-}e^{-i\left(2\omega_{p}-\omega_{s}\right)t}$$

Equation 5 demonstrates that, in addition to the pump frequency ω_*p*_ and the Stokes frequency ω_*s*_, a four-wave-mixing (FWM) field with frequency 2ω_*p*_ – ω_*s*_ is generated in this optomechanical cavity. Physically, pump photons (ω_*p*_) and Stokes photons (ω_*s*_) coherently drive molecular vibrations at the frequency ω_*v*_ = ω_*p*_ – ω_*s*_, and the blue-shifted anti-Stokes photons (ω_*as*_ = ω_*p*_ + ω_*v*_) can be generated by probing the excited vibrations with pump photons (ω_*p*_). A detailed energy-level diagram illustrating the CARS process is shown in Figure 1(b). Such phase matching can also emerge from the pump–probe spectra for molecular polaritons, as shown in recent progress.^62^

To investigate the CARS signal, we needed to detect the output field of the cavity mode. Using the ansatz, the output field can be expressed as ⟨*a*_*out*_⟩ = ⟨*a*_*out*_⟩_*ss*_ *e* ^–*iω*_*p*_ *t*^ + *a*_*out*,+_ *e* ^–*iω*_*s*_ *t*^ + *a*_*out*,–_ *e* ^–*i*(2ω_*p*_ – ω_*s*_)*t*^, where ⟨*a*_*out*_⟩_*ss*_, *a*_*out*,+_, and *a*_*out*,–_ are the responses at the pump frequency ω_*p*_, the Stokes frequency ω_*s*_, and the anti-Stokes frequency 2ω_*p*_ – ω_*s*_ of the output field, respectively. With the input–output relation^63^ $a_{\mathrm{out}}+\varepsilon_{p}/\sqrt{2\kappa }+\varepsilon_{s}e^{-i\delta_{s}t}/\sqrt{2\kappa }=\sqrt{2\kappa }a$ , the output signals are obtained as $a_{\mathrm{out},+}=\sqrt{2\kappa }a_{+}-\varepsilon_{s}/\sqrt{2\kappa }$ and $a_{\mathrm{out},-}=\sqrt{2\kappa }a_{-}$ .

To achieve a strong CARS signal, we consider the red-detuned resonance case (Δ = ω_*v*_); see Figure 1(c). Consequently, the relative intensities of generated anti-Stokes and Stokes fields, in term of the input Stokes field, can be obtained as^53^

$$I_{as}={\left|t_{as}\right|}^{2}={\left|\frac{a_{\mathrm{out},-}}{\varepsilon_{s}/\sqrt{2\kappa }}\right|}^{2}$$ 6a

6b $$I_{s}={\left|t_{s}\right|}^{2}-1={\left|\frac{a_{\mathrm{out},+}}{\varepsilon_{s}/\sqrt{2\kappa }}\right|}^{2}-1$$

where *t*_*as*_ = 4*iκ G* ^2^ *Nω*_*v*_/*C*(δ_*s*_), *t*_*s*_ = 2*iκ*{2|*G*|^2^ *Nω*_*v*_ – [ω_*v*_^2^ + (γ – *iδ*_*s*_)^2^]­(ω_*v*_ + δ_*s*_ + *iκ*)}/C*­(δ_*s*_) – 1, and *C*(δ_*s*_) = [(δ_*s*_ – *iγ*)^2^ – ω_*v*_^2^]­[(δ_*s*_ – *iκ*)^2^ – ω_*v*_^2^] – 4|*G*|^2^ *Nω*_*v*_^2^. Equations 6 indicate that *I*_*as*_ and *I*_*s*_ depend on κ and γ, while remaining independent of temperature. This is the intrinsic nature of the CARS process, which ensures stable signal generation across varying thermal environments, minimizing the need for active temperature control. It enhances measurement reliability in fluctuating environments and simplifies calibration and modeling, thereby improving the robustness of CARS for real-time quantum sensing. Note that the above analysis is based on an idealized model; in practice, both κ and γ generally exhibit temperature dependence. Additionally, a detailed discussion of the nonresonant background in CARS is provided in Supporting Information.^55^ The results show that the nonresonant contribution is negligible, allowing us to focus on the resonant components of the CARS signal, which capture the key spectral features of interest.

In Figure 2 we show *I*_*as*_ and *I*_*s*_ as functions of δ_*s*_ and *ε*_*p*_. The parameters are set to^35–38,50–52^ ω_*v*_/2π = 10 THz, κ/2π = 10 THz, γ/2π = 0.02 THz, *g*/2π = 10 GHz, and *N* = 100. The results show that the maximum intensities of anti-Stokes and Stokes scattering are located around δ_*s*_ = −ω_*v*_. Notably, as *ε*_*p*_ increases, the maximum intensity rises, while the vibrational resonance frequency undergoes an obvious shift. The underlying physical mechanism is as follows. The simultaneous presence of the pump and Stokes fields generates a radiation pressure force oscillating at the frequency δ_*s*_, and when δ_*s*_ approaches the vibrational frequency ω_*v*_, the molecule undergoes coherent oscillations, leading to significant enhancement of both anti-Stokes and Stokes intensities. Moreover, increasing *ε*_*p*_ enhances the effective coupling strength *G*, thereby boosting the signal intensity. The increased *G* also amplifies the optical spring effect, resulting in a substantial shift of the vibrational frequency. This particular attribute is of great significance for achieving room-temperature quantum precision measurement and quantum sensing.^64^

**Figure 2:**
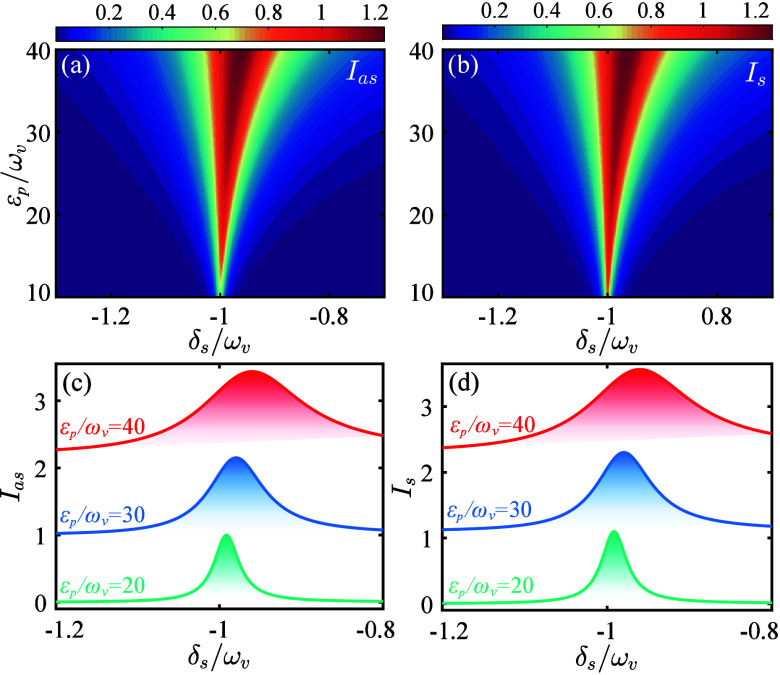
Relative intensity (a) *I*_*as*_ and (b) *I*_*s*_ versus detuning δ_*s*_/ω_*v*_ and pump amplitude *ε*_*p*_/ω_*v*_. (c) *I*_*as*_ and (d) *I*_*s*_ versus δ_*s*_/ω_*v*_ for different *ε*_*p*_. Other parameters used are γ/ω_*v*_ = 0.002, κ/ω_*v*_ = 1, *g*/ω_*v*_ = 0.001, Δ/ω_*v*_ = 1, and *N* = 100.

Other crucial parameters that determine the CARS signal include κ and γ. In Figure 3(a), we plot *I*_*as*_ (when δ_*s*_ = −ω_*v*_) as functions of κ and γ. The results show nonmonotonic behavior that first increases and then decreases with κ, and larger peak values can be achieved for smaller γ. Physically, moderate cavity dissipation facilitates photon output, thereby enhancing the output signal intensity. However, excessive dissipation significantly suppresses *G*, weakening the FWM process and reducing the signal output.^55^ Therefore, precise control of dissipation is crucial for amplifying the CARS response, ultimately improving both the sensitivity and spectral resolution in practical applications.

**Figure 3:**
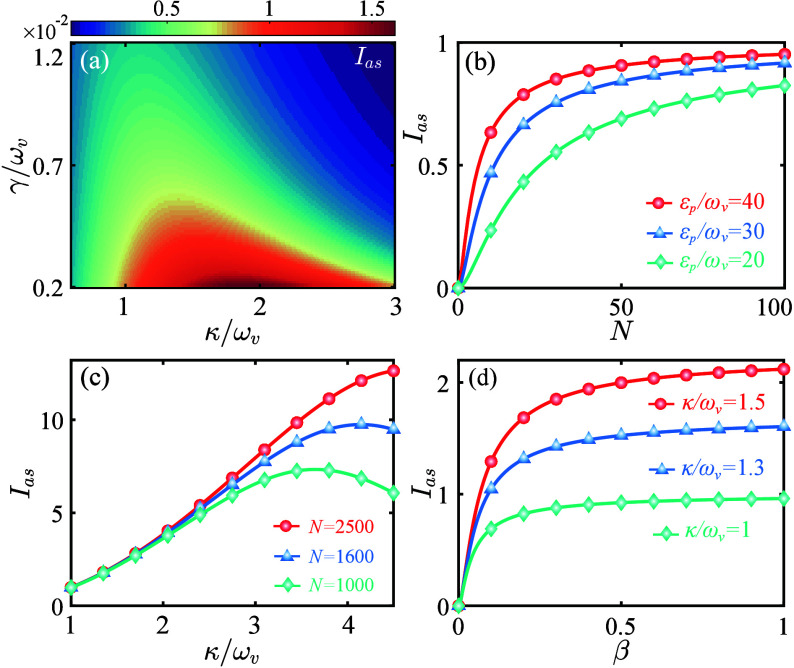
(a) Relative intensity *I*_*as*_ versus decay rates κ/ω_*v*_ and γ/ω_*v*_ when *ε*_*p*_/ω_*v*_ = 20. (b) *I*_*as*_ versus the number of molecules *N* for different *ε*_*p*_. (c) *I*_*as*_ versus κ/ω_*v*_ for different *N* values when *ε*_*p*_/ω_*v*_ = 20. (d) *I*_*as*_ versus steady-state mean value β (⟨*b*⟩_*ss*_) for different κ. Here, δ_*s*_ = −ω_*v*_ and other parameters are the same as those in Figure 2.

To explore the influence of collective enhancement effects on *I*_*as*_, in Figure 3(b) we plot *I*_*as*_ versus the number *N*. The curves demonstrate a monotonic increase in *I*_*as*_ as *N* increases. This phenomenon is attributed to the collective molecule–cavity interaction, which essentially brings out an √*N* enhancement of *G*. As a result, the required pump power is effectively reduced, lowering the threshold by a factor of *N*.^38^ This capability is advantageous for nondestructive sample detection. Furthermore, as *N* becomes sufficiently large, *I*_*as*_ saturates toward a value close to unity. This saturation arises from the relation *I*_*as*_ ∝ |κ/ω_*v*_|^2^ in the large-*N* limit,^55^ under the condition κ/ω_*v*_ = 1. Physically, the saturation originates from the intrinsic limitation of phonon energy available for anti-Stokes scattering: once a dynamic equilibrium is established between phonon generation and dissipation, further increases in *G* no longer result in proportional enhancement of the anti-Stokes signal. Inspired by this phenomenon, we increase the number *N* and optimize the cavity decay rate κ, achieving a CARS intensity exceeding 10 times that of the input Stokes light [Figure 3(c)]. These results demonstrate that the proposed optomechanical scheme not only significantly enhances CARS intensity and spectral sensitivity but also reduces power requirements, thereby broadening the practical applications of CARS technology.

To further investigate the coherence properties of the CARS signal, we derive the relationship between the steady-state mean value β­(⟨*B*⟩_*ss*_/√*N*) and *I*_*as*_,

7 $$I_{as}={\left|\frac{4\kappa gN\left(\gamma +i\omega_{v}\right)\omega_{v}\beta }{4gN\left(i\gamma -\omega_{v}\right)\omega_{v}^{2}\beta +F\left(\gamma ,\kappa \right)}\right|}^{2}$$

where *F*(γ, κ) = *γκ*(*iκ* + 2ω_*v*_)­(*iγ* + 2ω_*v*_) . In Figure 3(d), we examine the dependence of *I*_*as*_ on β. The results show that the CARS signal vanishes when β = 0, but increases significantly as β grows, indicating that the CARS signal can be amplified with increasing coherence. This behavior arises from the fact that *I*_*as*_ is a monotonically increasing function of β, reflecting enhancement of the FWM process with greater vibrational coherence. These findings underscore a key feature of the CARS signal, i.e., its sensitivity to the coherence in the molecular ensemble.

In addition to CARS, SRS is another form of coherent Raman scattering that can be realized in the molecular optomechanical cavity. Here, SRS is induced through a pump field and cavity photons (acting as Stokes or anti-Stokes fields), which scatter off the molecular vibrations. When their frequency detuning matches the vibrational frequency, a coherent and constructive force is exerted on the vibrational mode, resulting in strongly driven coherent oscillations, analogous to the stimulated nature of conventional SRS.

By applying the Fourier transform to the operators, the linearized QLEs ([Disp-formula eq3]) (without the input Stokes field) are converted into a frequency domain. The corresponding output cavity field fluctuations are then expressed as *a*_*out*_(ω) = *U*_11_(ω) *a*_*in*_(ω) + *U*_12_(ω) *B*_*in*_(ω) + *U*_13_(ω) *a*_*in*_^†^(ω) + *U*_14_(ω) *B*_*in*_^†^(ω), where *o*_*in*_(ω) and *o*_*in*_^†^(ω) for *o* = *a*, *B* are the noise operators and *U*_1*l*_(ω) for *l* = 1–4 are frequency-dependent coefficients.^55^ The output spectrum of the cavity field is defined as^65^

8 $$\begin{array}{c} S_{\mathrm{out}}\left(\omega \right)=\int_{-\infty }^{+\infty }d\omega^{\prime }\left\langle a_{\mathrm{out}}^{†}\left(\omega^{\prime }\right)a_{\mathrm{out}}\left(\omega \right)\right\rangle \\ =\left|U_{13}\left(\omega \right){|}^{2}+\left(\mathrm{n̅}+1\right)\right|U_{14}\left(\omega \right){|}^{2}+\mathrm{n̅}|U_{12}\left(\omega \right){|}^{2} \end{array}$$

where *n̅* is the average thermal phonon number. Here the first term is relevant to the spontaneous emission of the input vacuum noise *a*_*in*_, and the next two terms are caused by thermal noise *B*_*in*_. Equation 8 shows the temperature dependence of output spectrum *S*_*ou*t_(ω) . Experimentally, *S*_*out*_(ω) is directly observable, with particular interest in the Stokes and anti-Stokes components. The anti-Stokes/Stokes intensity ratio under red- and blue-detuned resonance cases can be expressed, respectively, as

9a $$R_{as/s}=\frac{4|G{|}^{2}N\kappa \omega_{v}^{2}+F\left(\mathrm{n̅}\right)\gamma \left(4\omega_{v}^{2}+\kappa^{2}\right)}{4G^{2}N\kappa \omega_{v}^{2}+\left[F\left(\mathrm{n̅}\right)+4\omega_{v}^{2}\right]\gamma \kappa^{2}}$$

9b $$R_{\mathrm{as}/s}=\frac{4G^{2}N\kappa \omega_{v}^{2}+F\left(\mathrm{n̅}\right)\gamma \kappa^{2}}{4G^{2}N\kappa \omega_{v}^{2}+\left[F\left(\mathrm{n̅}\right)+4\omega_{v}^{2}\right]\gamma \left(4\omega_{v}^{2}+\kappa^{2}\right)}$$

where *F*(*n̅*) = (2*n̅* + 1)­γ^2^ + 4*n̅ω*_*v*_^2^. Equations 9 demonstrates that temperature and dissipations are the key factors affecting the ratio *R*_*as*/s_. Notably, the Stokes and anti-Stokes components in SRS exhibit a nonlinear dependence on the number *N*, offering advantages over traditional SRS.^55^

In Figure 4(a) and 4(b) we plot *S*_*out*_(ω) as a function of *ε*_*p*_/ω_*v*_ and the normalized frequency ω/ω_*v*_ when the system works in the zero temperature environment (*n̅* = 0). The results show that there are two peaks located around ω/ω_*v*_ = ±1, which correspond to the maximum intensities of Stokes scattering (ω – ω_*p*_ = −ω_*v*_) and anti-Stokes scattering (ω – ω_*p*_ = ω_*v*_), respectively. Furthermore, the Raman scattering intensity exhibits an increase with the augmentation of *ε*_*p*_, and the Stokes scattering intensity is much stronger than the anti-Stokes scattering intensity in both red- and blue-detuned cases. Physically, the intensities of Stokes scattering and anti-Stokes scattering are proportional to the populations of the vibrational ground and excited states, respectively. At zero temperature, the excited-state population is negligible, resulting in a significantly stronger Stokes signal compared to the anti-Stokes signal.

**Figure 4:**
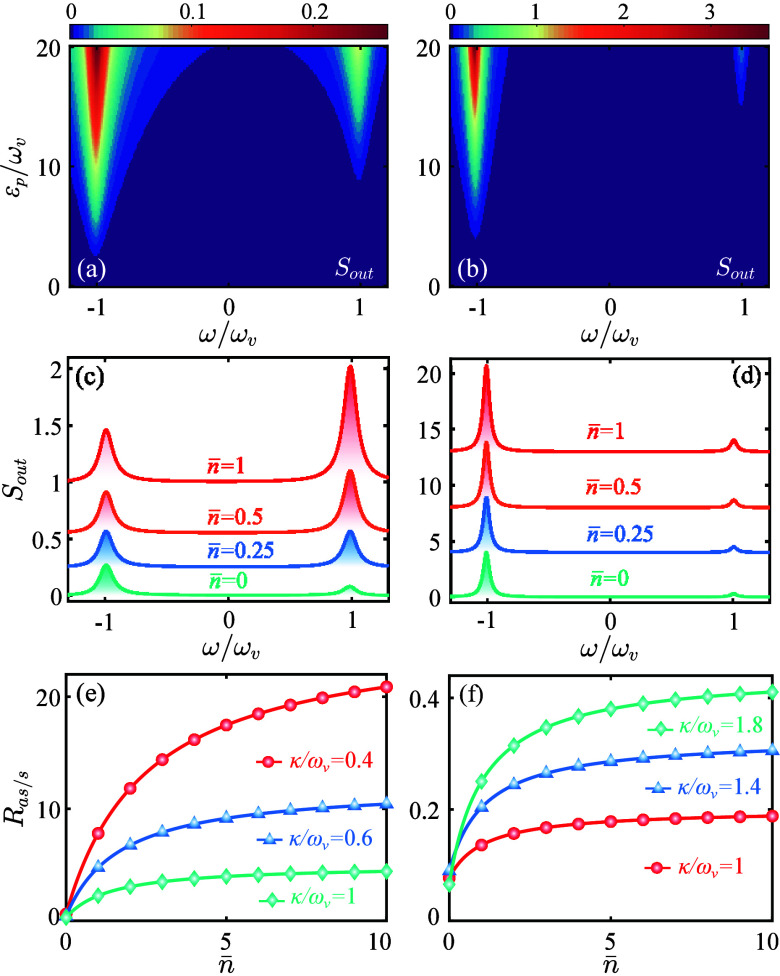
Output Raman spectrum *S*_*out*_(ω) versus *ε*_*p*_/ω_*v*_ and the normalized frequency ω/ω_*v*_ in (a) the red- and (b) blue-detuned resonance cases when *n̅* = 0. *S*_*out*_(ω) versus ω/ω_*v*_ for different thermal phonon number *n̅* in the (c) red- and (d) blue-detuned cases when *ε*_*p*_/ω_*v*_ = 20. Anti-Stokes/Stokes intensity ratio *R*_*as*/s_ versus *n̅* for different κ values in the (e) red- and (f) blue-detuned cases when *ε*_*p*_/ω_*v*_ = 20. Here γ/ω_*v*_ = 0.05 and other parameters are the same as those in Figure 2.

To investigate the dependence of Raman scattering on the system temperature, in Figures 4(c) and 4(d) we plot *S*_*out*_(ω) versus ω/ω_*v*_ and *n̅*. The results show that the increase of temperature is conducive to the enhancement of Raman scattering, and the intensity of anti-Stokes (Stokes) scattering increases much faster than that of Stokes (anti-Stokes) scattering in the red- (blue-) detuned case. Remarkably, we uncover an intriguing phenomenon that defies intuition: in the red-detuned case, an unexpected equivalence of the anti-Stokes and Stokes intensities is observed at room temperature (*n̅* = 0.25 corresponds to *T* ≈ 300 K). This phenomenon can be attributed to the Purcell effect. In the red-detuned case, the frequency of generated anti-Stokes photons (ω_*p*_ + ω_*v*_) matches the cavity resonance ω_*c*_ [see Figure 1(d)]. Consequently, the optical cavity significantly enhances the emission rate of anti-Stokes photons, increasing their generation probability and resulting in a pronounced amplification of the anti-Stokes scattering process. Moreover, at an elevated temperature, the thermal population of vibrational modes rises, enhancing the probability of phonon absorption by the pump field. This further strengthens the Purcell effect, leading to an additional enhancement of the anti-Stokes scattering intensity. Conversely, under the blue-detuned case, the Stokes scattering is much stronger than the anti-Stokes scattering, regardless of temperature variations.

To gain a deeper understanding of the temperature effect on the Raman scattering, in Figures 4(e) and 4(f) we plot anti-Stokes/Stokes intensity ratio *R*_*as*/s_ versus *n̅* for different κ. The results show that increasing the temperature can significantly increase the anti-Stokes/Stokes intensity ratio in both the red- and blue-detuned cases. Moreover, we find that in the red (blue) detuned case, the smaller (larger) the dissipation κ, the larger (smaller) the ratio *R*_*as*/s_. Indeed, in the red-detuned regime, a smaller κ enhances the effective coupling strength *G*, giving a stronger anti-Stokes resonance. The blue-detuned case follows the same way of understanding.

In summary, we developed quantum optomechanical Raman spectroscopy in which a molecular optomechanical cavity is driven by both a pump field and a Stokes field. Our work shows that increasing the pump strength greatly enhances the Raman cross section. The results revealed that the CARS signal is more than an order of magnitude stronger than the incident Stokes field. Moreover, we investigated the SRS process, noting the anti-Stokes resonance that is much lower than the Stokes resonance at zero temperature. Nonetheless, at finite temperature, our results demonstrated a significantly enhanced anti-Stokes resonance, which is anomalous. Our work would be insightful for developing efficient Raman detection of molecular structures at the few-photon level.

## Supplementary Material



## Notes

Detailed derivations of the intensity of the CARS signal, the nonresonant background, the output spectrum, and the molecular optomechanical Hamiltonian (ZIP)
