# Supplementary figures and images for: Generating Coherent Raman Scattering Using a Molecular Optomechanical Cavity
Source: Nano Lett. 2025 Nov 7;25(46):16372–8. doi: 10.1021/acs.nanolett.5c04075 (PMC12636078; doi:10.1021/acs.nanolett.5c04075)

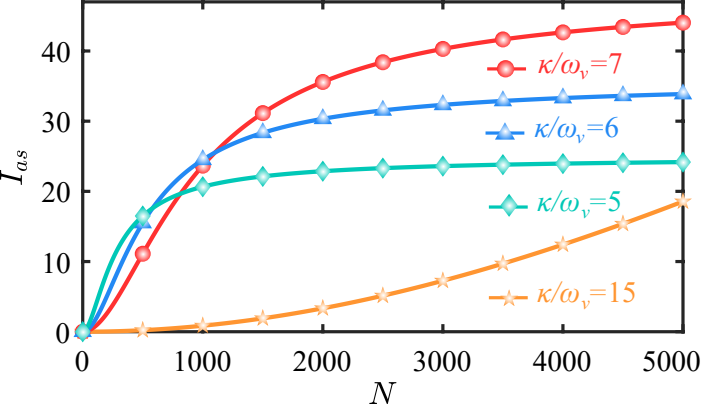

Supplement: Supplementary file 1 [file nl5c04075_si_001.zip › Supporting Information/FigS1.pdf]

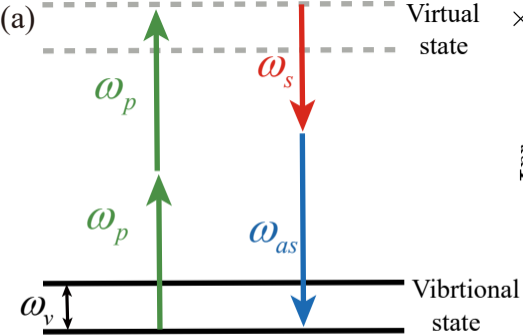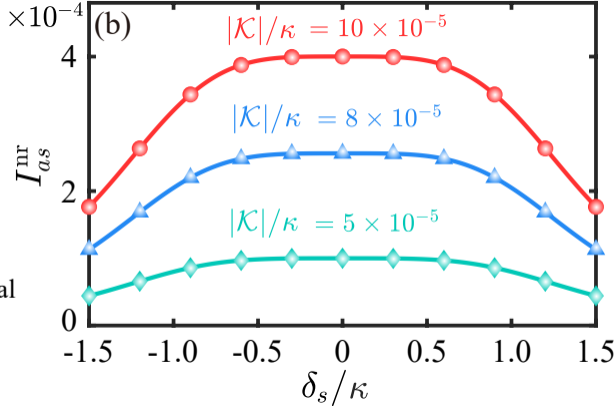

Supplement: Supplementary file 1 [file nl5c04075_si_001.zip › Supporting Information/FigS2.pdf]

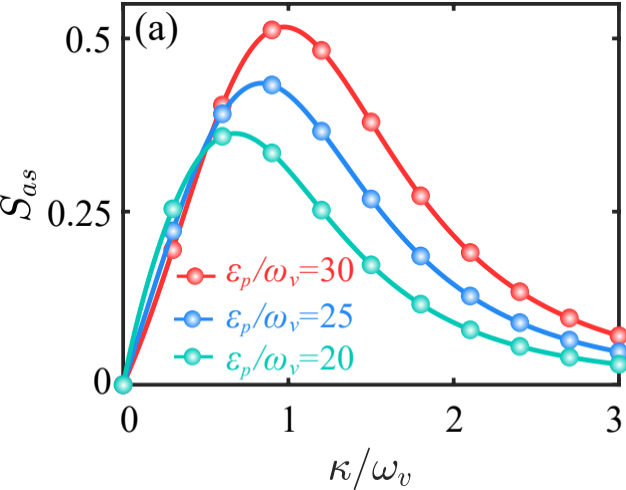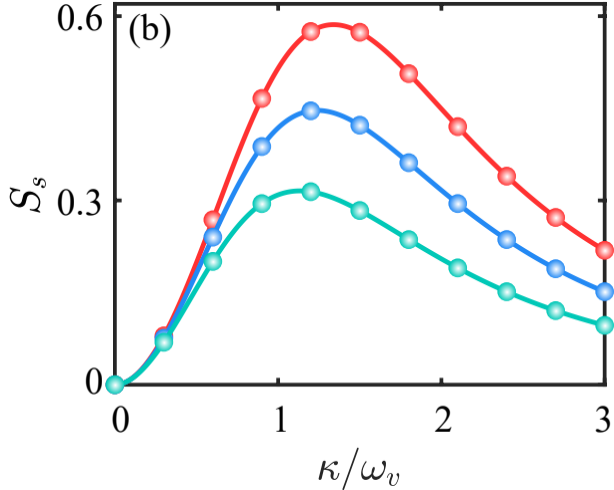

Supplement: Supplementary file 1 [file nl5c04075_si_001.zip › Supporting Information/FigS3.pdf]

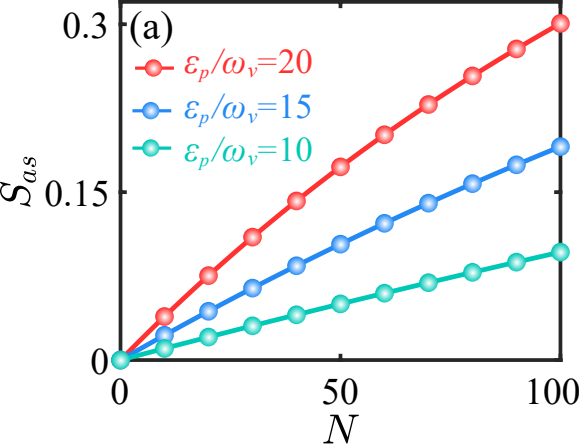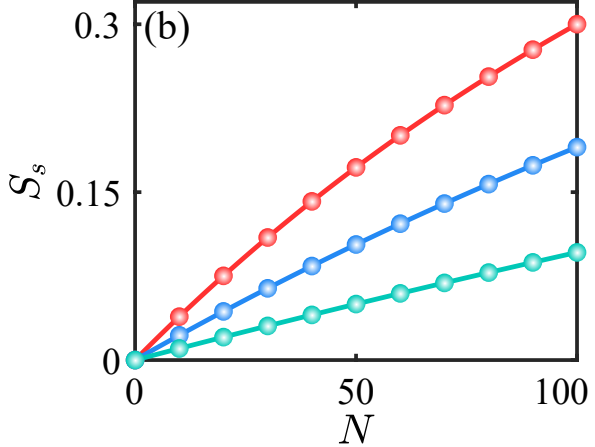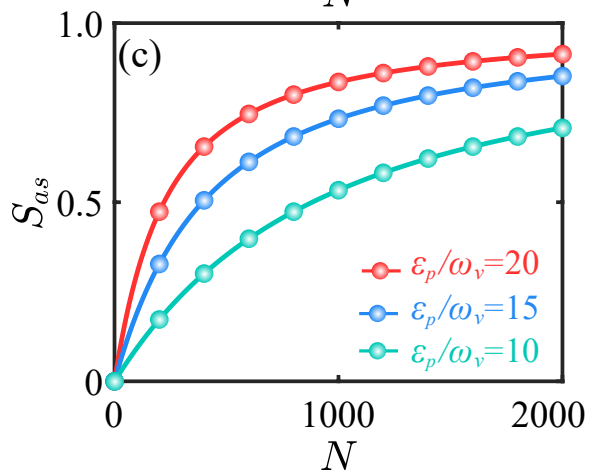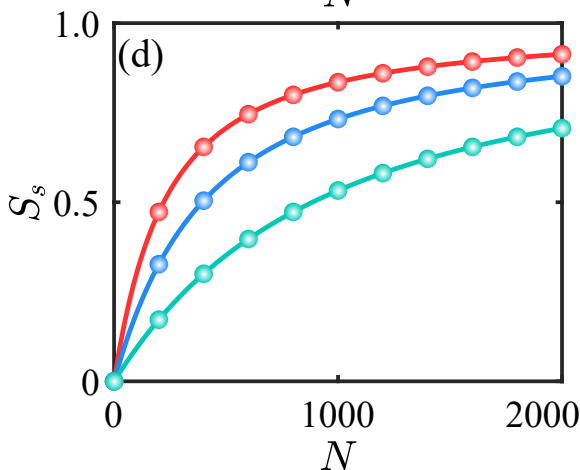

Supplement: Supplementary file 1 [file nl5c04075_si_001.zip › Supporting Information/FigS4.pdf]

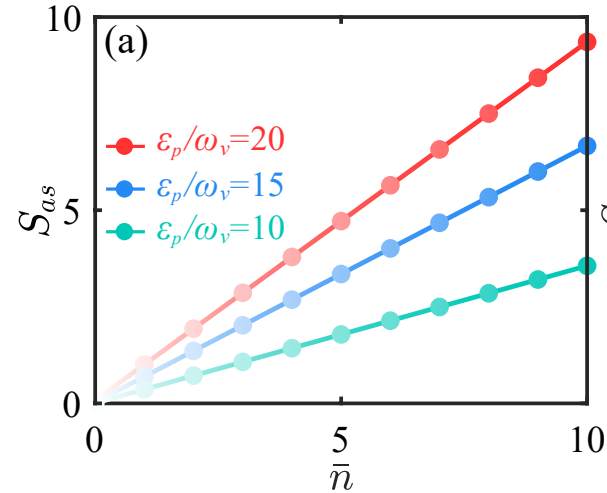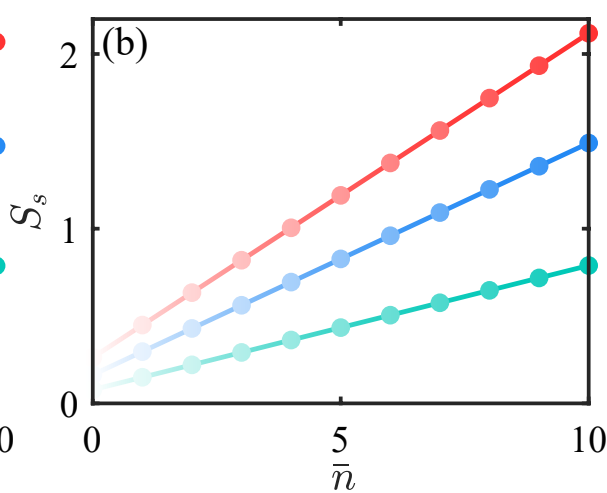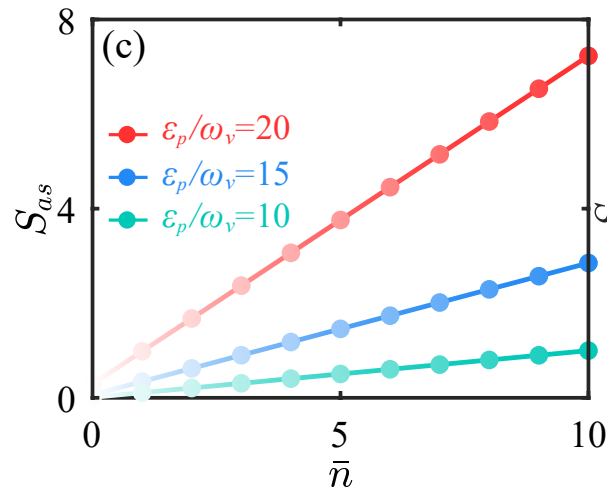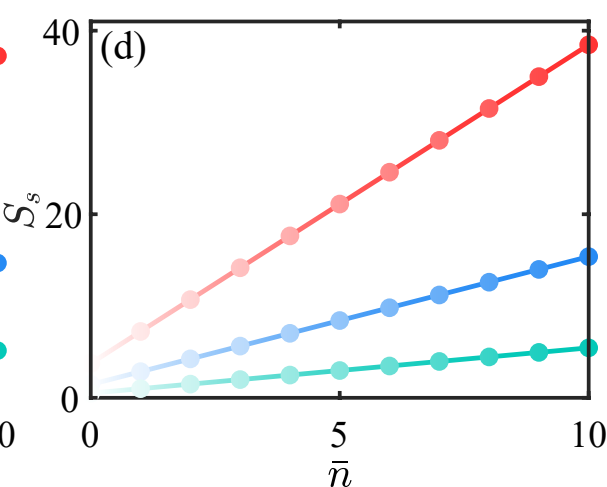

Supplement: Supplementary file 1 [file nl5c04075_si_001.zip › Supporting Information/FigS5.pdf]
